# To Evaluate the Severity, Distribution of Occlusal Tooth Wear and its Correlation with Bite Force in Young North Indian Adults

**DOI:** 10.2174/1745017901814010735

**Published:** 2018-09-28

**Authors:** Mandeep Kumar, Rashmi Verma, Mohit Bansal, Sunint Singh, Sharique Rehan, Virender Kumar, Dr. Simran

**Affiliations:** 1 Department of Prosthodontics, Rayat Bahra Dental College and Hospital, Mohali, Punjab, India; 2 Private Practice, Akalchet Multispeciality Dental Clinic, Sector-37 C, Chandigarh, India; 3 Department of Public Health Dentistry, Rayat Bahra Dental College & Hospital, Mohali, Punjab, India; 4 Department of Prosthodontics, Dr. Harvansh Singh Judge Institute of Dental Sciences and Hospital, Sector 25, Panjab University, Chandigarh, India; 5 Department of Conservative and Endodontics, Rayat Bahra Dental College & Hospital, Mohali, Punjab, India

**Keywords:** Bite force, Occlusal tooth wear, Young adults, Anterior teeth, Nonparametric test, Maxillary and mandibular casts

## Abstract

**Aim:**

To determine the severity and distribution of occlusal tooth wear among young North Indian adults and to evaluate the correlation of occlusal tooth wear with bite force.

**Materials and Methods:**

A total of 164 subjects were enrolled in the present study. Inclusion criteria included subjects with age range of 25-40 years having a full complement of natural dentition (excluding third molars), with no history of orthodontic treatment, FPD and trauma. Maxillary and mandibular casts of each subject were taken. Tooth wear score of anterior and posterior teeth of both the arches was calculated using a five-point (0 to 4) ordinal scoring system. The calculated tooth wear scores were then compared with data concerning age, sex, number of daily meals, vegetarian/non-vegetarian diet, Group function/Canine guided occlusion and bite force. Nonparametric (Mann-Whitney) test was used to determine the relationship between various factors and occlusal tooth wear. Spearman’s correlation coefficient was used to determine the correlation between tooth wear and bite force.

**Results:**

After applying statistical analysis to the data collected, total tooth wear score of the whole sample was 30.07 ± 6.39. Anterior teeth had significantly higher wear score than posteriors (*P* < 0.01). Males showed significantly higher (*P* < 0.001) tooth wear in both arches factors such as bite force and age showed significant correlation with tooth wear (*P*=0.000), however, the number of meals taken per day did not show any significant correlation. Higher tooth wear loss was seen in non vegetarian dietary pattern but it was statistically insignificant. It was also found that Group function occlusion showed significantly higher mean tooth wear loss 45.76 ± 9.19 as compared to Canine guided occlusion 26.37 ± 10.68 (*P*=0.000).

## 1 INTRODUCTION

Occlusal wear results from the loss of substance on opposing occlusal units or surfaces as a result of attrition or abrasion. Attrition is defined as the mechanical wear resulting from mastication or parafunction, limited to contacting surfaces of the teeth whereas, abrasion is the wearing away of a substance or structure through some unusual or abnormal mechanical process or by causes other than mastication [1]. Physiologically, attrition or wear of the occlusal surface of teeth may not be normal, but is also necessary for function [2]. Abrasion, erosion can also attribute to loss of tooth surface, but occlusal wear loss occurs at an ultrastructural level and is a physiological process. Many factors such as diet, clenching, bruxism and bite force are also associated with attrition [3]. Attrition is clinically manifested as small polished facets on the cusp or incisal edge or on the ridge to slight flattening or more over to the exposed dentine surface attrition [4]. 

Tooth wear (attrition, erosion and abrasion) is perceived internationally as a growing problem. For the comparison, diagnosis & classification of loss of dental hard tissues, many clinical and epidemiological studies have been developed [5]. Confusion has further generated in the literature, as the majority of researchers, in their attempts to quantify the amount of tooth tissue loss due to tooth wear, have historically concentrated on one etiology only, and these indices tend to be surface limited [6]. Often, the wear patterns described do not appear to reflect the etiology suggested, and this relates to lack of uniformity with tooth wear terminology and translation errors. Many diagnostic indices do not properly reflect the morphological defects, and there is little international standardization. All of these factors complicate the comparison of data and evaluation of the efficacy of preventive and therapeutic measures [7, 8]. The limitation of tooth wear surveys is the definition of the condition and parameter of measure. Loss of hard tissues of tooth irrespective of the surface causes one or more complications, but occlusal surface loss commands our attention for many reasons like effect on TMJ, masticatory efficiency; in extreme cases with loss of occlusal enamel, attrition can progress to dentine which has inferior resistance than enamel. In continuation to this, the pulpal involvement may become the associated problem [9]. 

## 2 MATERIALS AND METHODS

The present study was conducted on 164 subjects, irrespective of sex (99 males and 65 females), attending the OPD of different private clinics of North India. Inclusion criteria included subjects with age range of 20-40 years (with mean age 30.07 ± 6.390), having a full complement of natural dentition (excluding the third molar), with no history of orthodontic treatment, FPD and trauma. A detailed undersigned performa of the present study was introduced to all subjects enrolled in the study.

The sample size was estimated based on the pilot study done on 20 subjects. Estimating the prevalence to be 93% with 5% error and 95% confidence interval the sample size was determined. Both maxillary and mandibular anterior and posterior teeth were assessed clinically, using a five-point (0 to 4) ordinal scoring system (Table **1**) in which each tooth was given a score describing the severity of tooth wear [10]. Intraexaminer reliability was also determined and its Cronbach alpha (α) was found to be 0.83. Intraoral photographs of occlusal surfaces of both the arches; and maxillary and mandibular casts (Kalabhai Type III Dental stone) were also taken for each subject, to assist in the evaluation of the occlusal tooth wear.

**Fig. (2):**
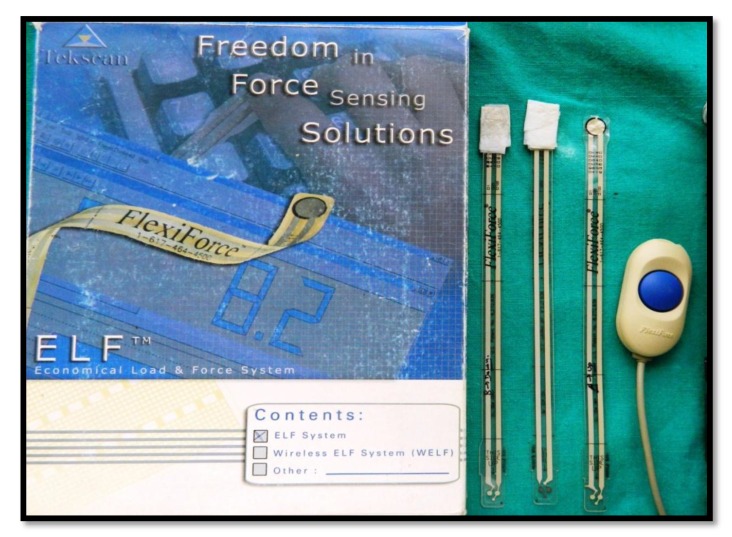
Elf force acquisition system with flexiforce sensors and USB attachment.

For measuring bite force, Bitometer (Tekscan, Flexiforce Sensors and ELF Data Acquisition System, USA, as shown in Fig. (**1**) was used. Maximum bite force measurements on the Flexiforce sensors in molar area were recorded (Fig. **2**). Three readings were taken and the highest value was selected. For eccentric relation evaluation, all subjects were guided for right and left movement of the mandible. Intraoral pictures were taken for all subjects to evaluate between canine guided or group function occlusion. A detailed questionnaire was produced for all subjects for evaluation of the total number of meals per day and for vegetarian/ nonvegetarian diet.

The tooth wear scores were compared with data concerning age, sex, bite force, Group function/Canine guided occlusion, number of daily meals, vegetarian/non-vegetarian diet, to determine specific correlates of tooth wear.

Nonparametric (Mann-Whitney) test was used to determine the relationship between various factors and occlusal tooth wear and Spearman’s correlation coefficient was used to determine the correlation between tooth wear and bite force.

## 3 RESULTS

Total tooth wear of the whole sample was 30.07 ± 6.39. Mean total tooth wear score for anteriors and posteriors was 18.77 ± 6.58 and 15.48 ± 8.09 respectively, which showed the anteriors had significantly higher wear score than the posteriors (*P* < 0.01) ( Table **2**). 

Comparing maxillary and mandibular arches individually (Table **3**), it was found that in mandibular arch, the anteriors had shown significantly higher tooth wear than posteriors, with a mean value of 12.74 ± 4.20 and 7.44 ± 3.99 respectively (*P* < 0.001). But, in maxillary arch, posterior teeth had shown significantly higher tooth wear than the anteriors, with a mean value of 8.04 ± 4.23 and 6.03 ± 3.50 respectively (*P*<0.001). Comparing the anteriors and the posteriors collectively of both the arches, it was found that mandibular the anteriors had shown more attrition than maxillary the anteriors whereas maxillary posteriors had shown more attrition than mandibular posteriors (*P*<0.001).The correlation coefficient of tooth wear in all quadrants is mentioned in the Table **4** which showed that tooth wear score had a significant correlation in all the quadrants (*P*=0.000).

The mean values of tooth wear score for both the sexes are mentioned in Table **5**. Males showed significantly higher tooth wear in both arches (both in the anteriors and the posteriors) with *P*< 0.001, except in maxillary anteriors. On comparing bite force for both males and females (Table **6**), it was found that mean bite force of males was significantly greater than females, with a mean value of 617.03 ± 42.98 and 507.98 ± 45.95 respectively (*P* < 0.001).

The correlation coefficient of tooth wear score with bite force, age and meals per day is mentioned in Table **7**. Bite force and age showed significant correlation with tooth wear (*P*=0.000), whereas the number of meals taken per day did not show any significant correlation. Mean values of tooth wear for vegetarians and non-vegetarians were found to be 32.76 ± 13.75 and 35.12 ± 13.95 respectively, which showed higher tooth wear loss in non-vegetarian dietary pattern (Table **8**). On comparing tooth wear for both the occlusal schemes (Table **8**), it was found that group function occlusal scheme showed higher mean tooth wear loss 45.76 ± 9.19 as compared to canine guided occlusal scheme 26.37 ± 10.68 (*P*=0.000).

## 4 DISCUSSION

Physiologically, occlusal surface loss is compensated by passive eruption of teeth. Therefore, the decrease in occlusal vertical dimension or increase in freeway space is not always a result of occlusal tooth wear. The estimated normal occlusal enamel loss from natural wear is about 65µm/year [4]. Pintado *et al.*, in his 2 years follow up study, found that 10.7 microns of occlusal surface loss occurred only in the first year of his study [9]. Patient’s adaptive ability to change in vertical dimension in dentate condition is highly considerable. Developmental defects in enamel and dentin formation such as Amelogenesis Imperfecta and Dentinogenesis Imperfecta should be differentiated from regressive changes in the dental hard tissue caused by attrition. Measurement of the amount of tooth wear is essential and plays important role in maintaining the integrity of the stomatognathic system and to know at what stage of tooth wear, one should start the restorative treatment of worn dentition [10]. As advanced attrition will have an adverse effect on normal balance and function of the masticatory system, TMJ and vertical facial dimensions, therefore, occlusal rehabilitation is mandatory in such cases [11]. 

In the present study, occlusal tooth wear was measured by using modified criteria. The average tooth wear score of total sample was found to be 30.07 ± 6.39. It had also been found that anterior teeth had shown significant attrition than posterior teeth (*p*<0.01), which correspond with the findings of Hooper SM *et al.,* Hugoson A *et al*., Johansson *et al*., [12-14]. Mandibular anterior teeth showed maximum tooth wear followed by maxillary posterior teeth, mandibular posteriors, maxillary anteriors. Canines had shown highest attrition, which can be well supported by the study by Pigno that due to increased force that is exerted on canines during biting and lateral jaw movements in individuals with increased bite force and a canine guidance occlusion [14, 15]. 

Many factors contribute to the attrition *e.g.* bite force, age, sex, occlusal scheme, psychosocial status, dietary pattern. Bite force is described as the force applied by the masticatory muscles to produce strain on the mandibular teeth against maxillary teeth [16]. Literature review supported by many studies showed that males present higher maximum bite force due to higher muscle strength [17]. In the present study, mean maximum bite force for men was calculated as 606.8N ± 45.5 and for females, it was 486.2N ± 41.7, which corresponds to that reported by Singh who reported 650.47 and 543N in males and females respectively [18]. A positive correlation between the masseter muscle thickness and the bite force was found by Bakke *et al.*, and Raadsheer *et al.*, [19, 20]. The literature regarding the relationship between bite force and tooth wear is inconclusive, as being studied by many authors [15, 21] ,while Johansson [22] demonstrated a significant correlation between submaximal bite force and tooth wear. In the present study, a significant difference was found between males and females, with males having higher tooth wear and bite force (*P* < 0.001), this foregoing contention is supported by additional finding of Hugoson [13] Johansson [22] who in their studies found some evidence of gender effect on dental attrition, with greater attrition reported in males than in females.

In the present study, Tooth wear had also shown a significant correlation (r = 0.333, *P*=0.000) with age. Pollman *et al*., also found that elderly people showed significant tooth wear than younger ones [23]. Spijker found in his prospective study that with advancement in the age the tooth wear increases from 3% (at 20 years of age) to 17% (at 70 years of age) [24]. However, a number of younger subjects showed significant attrition, which resulted from other factors that contribute to the extent of occlusal tooth wear.

Significant tooth wear was found in subjects with group function as compared to canine guided occlusion (*P* = 0.000) [25]. Regarding dietary pattern, no significant correlation was found between tooth wear and vegetarians/ nonvegetarians. Meals per day also showed no significant correlation with occlusal tooth wear. This may be attributed to the changes in the dietary habits and pattern [26]. 

The major drawback of our study is the criteria used for assessment of tooth wear is subjective based on individual perception. More recent studies can be done including the quantitative measurement of tooth wear score.

## CONCLUSION

Factors which found to be associated with occlusal tooth wear were age, sex, bite force, Group function or canine guided occlusal scheme, however dietary pattern as well as the number of meals per day did not show any correlation.males showed significantly greater tooth wear than females. We found that tooth wear had a significant correlation with age. Group function had shown significant occlusal tooth wear than canine guided occlusion.

## Figures and Tables

**Fig. (1):**
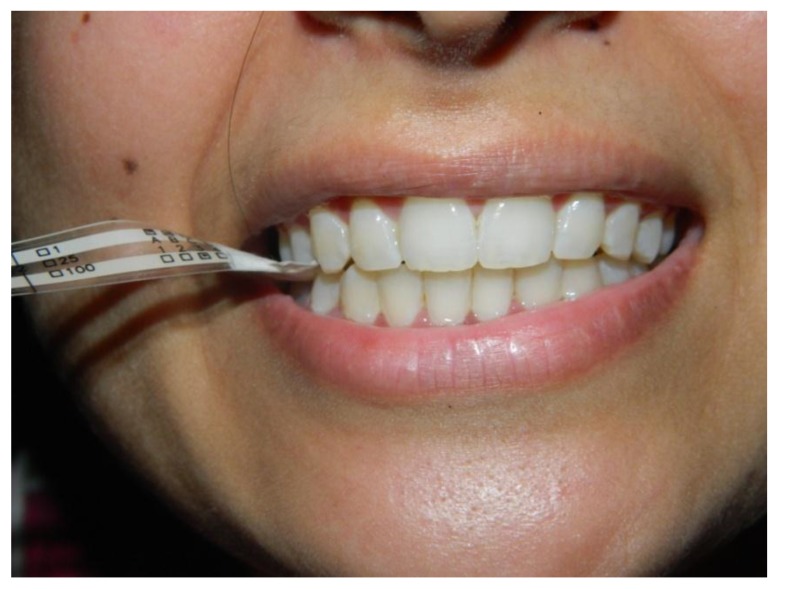
Bitometer in patient’s mouth.

**Table 1:** Ordinal scoring system.

Score	Surface	Criterion
0	OI	No loss of enamel surface characteristics
1	OI	Loss of enamel surface characteristics
2	O I	Enamel loss just exposing dentine <1/3 of the surface Enamel loss just exposing dentine
3	O I	Enamel loss just exposing dentine >1/3 of the surface Enamel loss and substantial dental loss but no pulp exposure
4	O I	Complete enamel loss, or pulp exposure or 2^0^ dental exposure Pulp exposure or 2^0^ dental exposure

O=Occlusal; I=Incisal

**Table 2:** Mean values for tooth wear of anterior and posteriors and their comparison.

**-**	**Tooth Wear Score** **(Mean ± S.D)**
**Total Tooth Wear Score**	30.07 ± 6.39
**Anteriors**	18.77 ± 6.58
**Posteriors**	15.48 ± 8.09
**Z value**	-7.659
***P* value**	<0.01

**Table 3:** Mean values for tooth wear score for maxillary and mandibular anteriors and posteriors and their comparison.

**Tooth Wear Score**		**Max Ant**		**Mand Ant**		**Max Post**		**Mand Post**	
**Mean ± S.D.**		6.03 **±** 3.505		12.74 **±** 4.206		8.04 **±** 4.236		7.44 **±** 3.993	
**Median**		5.00		12.00		8.00		8.00	
**-**		**Max Post *Vs* Max Ant**		**Mand Post *Vs* Mand Ant**		**Mand Ant *Vs* Max Ant**		**Mand Post *Vs* Max Post**	
**Z value**		-6.530		-10.554		10.888		-4.854	
***P* value**		<.001**		<.001**		<.001**		<.001**	

**Table 4:** Correlation coefficient of anteriors and posteriors of maxillary and mandibular arch with tooth wear score.

**Total Tooth Wear Score**	**Correlation Coefficient**	***P* value**
**Maxillary anteriors**	0.714	0.000
**Mandibular anteriors**	0.806	0.000
**Maxillary posteriors**	0.926	0.000
**Mandibular posteriors**	0.916	0.000

**Table 5:** Mean tooth wear score of males and females and their comparison.

**Sex**	**Total Tooth Wear Score**			
**-**	**Maxillary Anteriors**	**Mandibular Anteriors**	**Maxillary Posteriors**	**Mandibular Posteriors**
**Male**	6.78 ± 3.73	14.69 ± 3.52	9.52 ± 3.29	8.53 ± 3.46
**Female**	2.79 ± 4.89	9.78 ± 3.36	5.80 ± 4.55	5.78 ± 4.20
**Z value**	-3.118	-7.843	-6.474	-4.653
***P* value**	0.002	<0.001	<0.001	<0.001

**Table 6:** Mean bite force score of males and females and their comparison.

**Sex**	**Bite Force** **(Mean ± S.D)**
**Male**	617.03 ± 42.98
**Female**	507.98 ± 45.95
**Z value**	-9.977
***P* value**	<0.001

**Table 7:** Correlation coefficient of tooth wear score with bite force, meals per day and age.

**Total Score**	**Correlation Coefficient**	***P* value**
**Bite force**	0.732	0.000
**Meals per day**	0.101	0.197
**Age**	0.333	0.000

**Table 8:** Comparison of mean tooth wear score between vegetarian and non vegetarian and between Canine guided and Group function occlusion.

-	**Dietary Pattern**		**Occlusal Scheme**	
	**Vegetarian(n=66)**	**Non Vegetarian (n=98)**	**Canine Guided Occlusion**	**Group Function Occlusion**
**Tooth Wear Score** **(Mean ± S.D.)**	32.76 ± 13.75	35.12 ± 13.95	26.37 ± 10.68	45.76 ± 9.19
**Z value**	-0.984		-9.458	
***P* value**	0.325		0.000	
